# Quantitative Assessment Parameters of Peripapillary Regions with Branch Retinal Vein Occlusion by Using Optical Coherence Tomography Angiography

**DOI:** 10.1155/2022/9281630

**Published:** 2022-11-03

**Authors:** Shan Yin, Yanyan Cui, Wanzhen Jiao, Bojun Zhao

**Affiliations:** ^1^Department of Ophthalmology, Central Hospital Affiliated to Shandong First Medical University, 250014 Jinan, China; ^2^Department of Ophthalmology, Liaocheng People's Hospital, 252004 Liaocheng, China; ^3^Department of Ophthalmology, Shandong Provincial Hospital Affiliated to Shandong First Medical University, 250000 Jinan, China

## Abstract

**Purpose:**

To investigate the baseline parameters of peripapillary regions in both eyes of patients with unilateral branch retinal vein occlusion (BRVO) using optical coherence tomography angiography (OCTA) and their association with best-corrected visual acuity (BCVA).

**Methods:**

Forty-eight unilateral BRVO patients were enrolled. The 4.5 × 4.5 mm disc angiogram was acquired in the BRVO eyes and fellow eyes using the OCTA. Radial peripapillary capillary (RPC), whole vessel density, and retinal nerve fiber layer (RNFL) thickness in different regions and optic nerve head (ONH) analysis were automatically calculated. The partition includes the whole image, peripapillary, superior hemifield, inferior hemifield, eight equally divided sectors, and nine evenly divided square areas.

**Results:**

All vessel density and capillary vessel density in the whole and peripapillary regions of BRVO eyes were significantly lower than those in fellow eyes. The RNFL peripapillary thickness in BRVO eyes was significantly higher than those in fellow eyes. In eyes with supertemporal vein occlusion, all vessel density both in the superior hemifield and in the G12 region was significantly reduced compared with that in the fellow eyes. The capillary vessel density was significantly lower in the superior hemifield, superior temporal (ST), superior nasal (SN), and temporal superior (TS) areas than in the fellow eyes. The RNFL thickness in the NI, IN, TI, and TS sectors was significantly higher than in fellow eyes (all *P* < 0.05).

**Conclusions:**

OCTA provided quantitative information on peripapillary vascular density and RNFL thickness changes in BRVO. Branch retinal vein occlusion not only affects the blood vessel density in the macular area but also decreases the radial peripapillary capillaries. The capillary density is mainly affected in the affected hemifield but not in the unaffected hemifield.

## 1. Introduction

Branch retinal vein occlusion (BRVO) is considered the most common among RVOs, with an incidence of 0.5% to 1.2% [1]. Macular edema (ME) is a major complication of BRVO that results in significant visual impairment [1–3]. The combination of compression of veins at artery and vein crossings, degenerative changes within venous walls, and hypercoagulability may be the pathogenetic mechanism. Moreover, the extent of retinal ischemia determines the outcome of BRVO as a consequence of retinal and iris neovascularization [1]. Thus, a quantitative assessment of the blood flow of the retinal vascular system is important to predict the prognosis of BRVO.

Optical coherence tomography angiography (OCTA) is a noninvasive imaging technique that has great potential for use in the clinical setting. It can visualize the retinal vasculature in detail and provide a quantitative assessment of retinal blood vessels. Several researchers studied the macular vessel density in BRVO patients [4, 5]. However, there is a limited literature on regional analysis of peripapillary microvasculature in BRVO patients.

The radial peripapillary capillary (RPC) layer is a delicately organized vasculature radiating from the optic nerve head (ONH) that supplies the retinal nerve fibers surrounding the ONH [6, 7]. The RPCs are the most superficial layer of capillaries lying in the inner part of the RNFL, and they run along the paths of the major retinal vessels up to 4-5 mm from the ONH. Some researchers postulated that these capillaries, having long parallel paths and rare anastomoses, may be more vulnerable to increased intraocular pressure (IOP) or hemodynamic disorders, and thus may be affected selectively before other retinal capillary networks [8–10]. Two investigators evaluated the changes in peripapillary microvascular parameters in the fellow eyes of patients with unilateral RVO using OCTA and determined the relationships between peripapillary microvasculature and RNFL thickness [11, 12]. Due to the difference in pathogenesis between BRVO and CRVO, it is necessary to analyze the peripapillary microvasculature and RNFL thickness separately.

## 2. Methods

### 2.1. Patients

48 patients diagnosed with unilateral BRVO who were treated at the Department of Ophthalmology of Shandong Provincial Hospital Affiliated to Shandong First Medical University between July 2020 and October 2021 were enrolled in this retrospective observational study. A typical fundus examination and fundus photography were used to diagnose BRVO. Treatment-naïve, unilateral BRVO involving the macular area were included in this study. Their normal fellow eyes were used as normal controls. The exclusion criteria for this study were a history of ocular surgery, bilateral BRVO, onset duration >6 months, presence of systemic disease (except for hypertension), and presence of retinal diseases other than BRVO. OCTA images with low-quality and media opacity were also excluded.

All patients underwent a thorough ophthalmologic examination, including the best-corrected visual acuity (BCVA), IOP, slit-lamp biomicroscopy, indirect ophthalmoscopy, fundus photography, and OCTA (RTVue-XR Avanti, Optovue, Inc., Fremont, CA).

### 2.2. Image Acquisition

**Figure 1:**
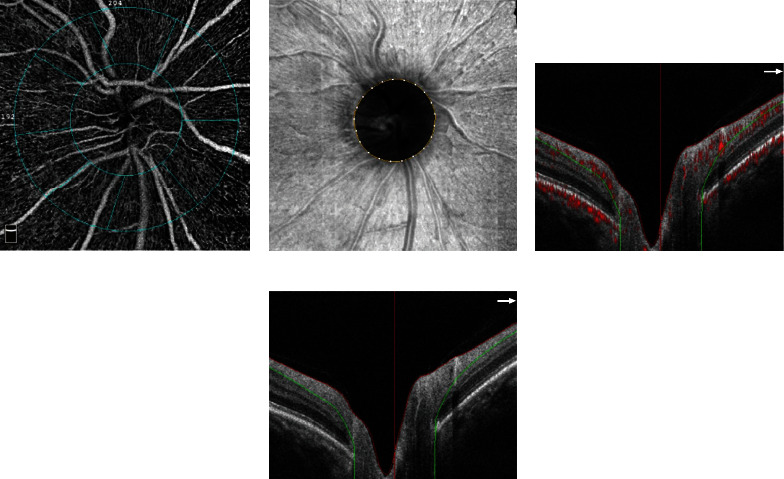
The OCTA image of optic disc in a BRVO patient. (a) An Angio image. (b) An en face image. (c) A horizontal B-scan. (d) A vertical B-scan.

**Figure 2:**
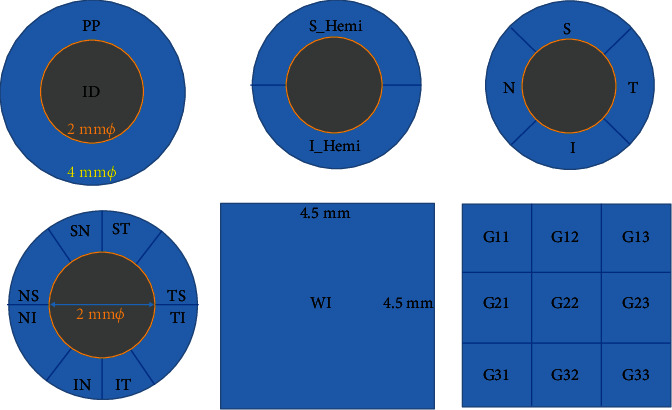
Peripapillary grid and whole image measurement areas. WI: whole image of 4.5 × 4.5 mm of Angio Disc scan; PP: peripapillary; ID: inside disc (grey area outlined by the yellow boundary); S-Hemi: superior hemifields; I-Hemi: inferior hemifields; S: superior quadrant; T: temporal quadrant; I: inferior quadrant; N: nasal quadrant; NS: nasal superior sector; NI: nasal inferior sector; IN: inferior nasal sector; IT: inferior temporal sector; TI: temporal inferior sector; TS: temporal superior sector; ST: superior temporal sector; SN: superior nasal sector.

A rectangle scan of 4.5 × 4.5 mm centered on ONH was obtained for BRVO eyes and fellow eyes with the Angiovue OCTA system using Angio-Disc mode (like Figure 1). The software can automatically measure the vessel density and thickness of the RNFL within Angio Disc scan measurement areas, and ONH analysis, including cup/disc area ratio, cup/disc vertical ratio, cup/disc horizontal ratio, rim area, and cup volume.  Figure 2 provides a schematic presentation of the vessel density and RNFL thickness measurement areas and parameters nomenclature based on the whole image and Garway-Heath based peripapillary grid.

### 2.3. Statistical Analysis

All statistical analysis was performed using SPSS (SPSS for Mac, version 23.0; IBM/SPSS, Chicago, IL, USA). The data are presented as mean ± standard deviation. The BCVA measured on an international standard logarithmic visual acuity chart was converted to the logarithm of the minimum angle of resolution (logMAR) prior to statistical analysis. Paired *t*-test was used to compare the vessel density (VD) and RNFL thickness between BRVO eyes and normal fellow eyes in each region. Additionally, to better compare VD and RNFL thickness in different regions, BRVO patients were divided into the supertemporal BRVO group and inferotemporal BRVO group. The Pearson correlation coefficient was used to evaluate the correlations between logMAR BCVA and OCTA parameters of disc.

## 3. Results

A total of 48 patients with unilateral BRVO were enrolled. The demographic and clinical characteristics of the participants are summarized in Table 1. The mean age was 54.0 ± 9.50 years. Among the 48 included patients, 45.8% were males and 54.2% were females. The mean BCVA of the BRVO eyes and the normal fellow eyes was 0.686 ± 0.424 and 0.131 ± 0.169, respectively, and this difference was statistically significant.

### 3.1. The OCTA Parameters of BRVO

The capillary vessel density in the whole and peripapillary regions were 49.18 ± 2.92%, 51.74 ± 3.45% in BRVO eyes. All vessel density in the whole and peripapillary regions were 55.87 ± 2.83%, 58.12 ± 3.36% in BRVO eyes. Those were significantly lower than those in fellow eyes (all *P* < 0.05). (Table 2) The large peripapillary vessel density (calculated by subtracting capillaries from all vessel density) in the whole and peripapillary regions were 6.69 ± 0.69%, 6.37 ± 0.76% in BRVO eyes. Those were significantly higher than those in fellow eyes (all *P* < 0.05). (Table 2) The RNFL thickness of the peripapillary in BRVO eyes was significantly higher than in fellow eyes (*P* < 0.05), (Table 2).

The ONH analysis including cup/disc area ratio, cup/disc vertical ratio, cup/disc horizontal ratio, rim area (mm^2^), and cup volume (mm^3^) was not statistically different between BRVO eyes and fellow eyes (Table 2).

### 3.2. The OCTA Parameters of Supertemporal BRVO

In eyes with supertemporal vein occlusion, we analysed all vessel in superior hemifield, inferior hemifield, G11, G12, G13, G21, G22, G23, G31, G32, and G33 areas. Among of those parameters, all vessel density in superior hemifield and G12 area of the affected eye were significantly lower than that of the fellow eyes. The large peripapillary blood vessel density in superior hemifield was significant higher compared with the fellow eyes (all *P* < 0.05), (Table 3).

We also detected the capillary vessel density and RNFL thickness in superior hemifield, inferior hemifield, quadrants of inferior, superior, temporal, nasal, and the sectors of inferior temporal, inferior nasal, superior temporal, superior nasal, nasal inferior, nasal superior, nasal superior, and nasal inferior. The capillary vessel density in superior hemifield, the quadrants of the superior, temporal, and the sectors of temporal superior, superior temporal, and superior nasal were significantly lower than that of fellow eyes. The RNFL thickness in superior hemifield, inferior hemifield, the quadrants of nasal, temporal, and the sectors of nasal inferior, inferior nasal, temporal inferior, and temporal superior were significantly higher than that of fellow eyes (all *P* < 0.05), (Tables 3 and 4).

### 3.3. The OCTA Parameters of Inferotemporal BRVO

We analysed the same parameters in eyes with inferotemporal BRVO as supertemporal BRVO. All vessel density in inferior hemifield and G32 area of inferotemporal BRVO were significant lower compared with the fellow eyes. The capillary vessel density in inferior hemifield, the quadrants of inferior, temporal, and the sectors of temporal inferior, inferior temporal, and inferior nasal were significantly lower than that in fellow eyes. The RNFL thickness in temporal quadrant and temporal inferior sectors were significantly higher than that in fellow eyes. The RNFL thickness in inferior nasal sectors was significantly lower than that in fellow eye (all *P* < 0.05), (Tables 5 and 6).

### 3.4. The Correlations between BCVA (logMAR) and OCTA Parameters

Because a small number of patients with inferotemporal BRVO were enrolled, we mainly analysed the correlations of BCVA and OCTA disc indicators in the eyes with supertemporal BRVO patients. The Pearson correlation analysis showed that all positive indexes we studied were not significantly associated with VA.

## 4. Discussion

In previous decades, fluorescein angiography (FA) was used in the evaluation of ONH perfusion. However, RPCs were difficult to be observed with conventional FA. RPC can be easily visualized, and vascular perfusion status can be quantified within minutes to OCTA. In addition, compared with laser Doppler flowmetry, OCTA is less affected by the absorbance and reflectance of disc tissue. It has been considered as a useful parameter for evaluating vascular dysfunction. In our study, the capillary density and all vessel density of the whole image and peripapillary in BRVO eyes were lower than those of fellow eyes. The changes in capillary density were consistent with the study of Chen et al. [13]. Their study only showed the density of capillaries. This indicates that the capillaries in the RNFL around the optic nerve were damaged, not just in the macula. The increase in the intravascular pressure due to BRVO leads to a wide range of reductions in capillary vascular density. In the research of Takahashi et al., the diameter of the occluded vein in BRVO patients at half-disc diameter away from the optic disc was smaller than healthy veins. And, the retinal blood flow in the occluded vein was lower than in the healthy veins [14]. We found that the density of the larger blood vessels in the whole image and peripapillary was higher compared to that of the fellow eyes. Therefore, we speculate that the change in the larger blood vessel density may be related to the arteries. Due to the compression of veins at AV crossings, venous blood flow decreased and arterial blood flow compensatory increased, and the increase in arterial blood flow was greater than the decrease in venous blood flow. In addition, the capillary vessel density showed the most significant statistical difference, indicating that BRVO caused the most damage to the capillaries around the optic disc.

To analyze the changes in vascular density in different areas around the optic disc of BRVO patients in more detail, we divided the enrolled patients into the supertemporal BRVO group and the inferotemporal BRVO group. In the supertemporal BRVO group, the damaged capillaries mainly occurred in the affected superior hemisphere, while they did not influence the unaffected inferior hemisphere. In the affected hemisphere, the capillary density of TS, ST, and SN sectors was statistically decreased compared to that of the fellow eyes, while the capillary density in the NS sector was not statistically different. Mainly because the NS sector was supplied by nasal retinal vessels, the occlusion of the supertemporal retinal vein did not influence this sector. The 4.5 × 4.5 mm area was divided into 9 equal parts to measure all vascular density of each area in eyes with supertemporal vein occlusion, only G12 showed significant difference compared with fellow eyes. This also indirectly proved that the capillary vessel density in the superior quadrant showed the most significant statistical difference. Chen et al. research found the inferior temporal sector in the supertemporal BRVO eyes was statistically difference compared to the contralateral eyes [13]. This difference may be explained by the fact that they included both treated and untreated patients. However, further follow-up is required.

Some researchers reported that RNFL thickness decreased in the fellow eyes of unilateral RVO patients [12, 15, 16]. Conversely, Altunel et al. [17] and Lim et al. [18] showed that the average RNFL thickness did not differ between RVO-unaffected eyes and the eyes of healthy controls. There is controversy about the RNFL thickness changes in the RVO-unaffected eyes compared with healthy eyes. We mainly studied the changes in RNFL thickness in BRVO eyes. Due to the swelling of the ONH, our research showed that in the supertemporal BRVO eyes, the RNFL thickness in both the affected superior and unaffected inferior hemifields was significantly higher than that of the fellow eyes. And, the difference was even more pronounced in the inferior hemisphere. We further analysed the RNFL thickness in the eight equally divided sectors of the affected eyes and fellow eyes in patients with supertemporal BRVO. There were statistical differences in the four sectors, three of which were in the inferior hemisphere. We hypothesize that the possible cause is that the fluid exudate by retinal blood vessels causes edema of RNFL in the inferior hemisphere due to gravity. In the inferotemporal BRVO eyes, only the thickness of RNFL in the sectors of IN and TI was statistically different from that in fellow eyes. The main reason may be that fewer patients were enrolled in the group. Further research is needed to determine whether inferotemporal branch vein occlusion will affect the unaffected hemisphere.

The correlation analysis of all positive parameters with the baseline BCVA found that they had no significant correlation. The ONH analysis indexes showed no difference between the affected eyes and the fellow eyes, so it can be considered that the changes in vascular density and RNFL thickness in peripapillary regions are not enough to affect visual activity, and the visual impairment of BRVO patients is mainly caused by the structural damage to the macular.

Some limitations remain in this research. There is likely a selection bias because of the sample size was limited. In addition, the analysis of OCTA parameters in patients with nasal BRVO is necessary, but the number of patients with nasal BRVO in our collection is very small and does not allow for statistical analysis. All patients enrolled are still being followed up and the data are being accumulated; a longitudinal study result will be analysed when all parameters collection is complete. BCVA is one aspect of evaluating visual function and may not fully reflect the impairment of visual function. Another limitation in our study was that the patients were not examined for visual fields, and it would also be very interesting to analysed the correlation between visual fields and OCTA parameters.

## 5. Conclusion

In conclusion, our study demonstrated that OCTA can provide high-resolution images and quantitative information about microvascular parameters of peripapillary vascular density in BRVO eyes. BRVO not only affects the blood vessel density in the macular area but also decreases the radial peripapillary capillaries. The capillary density is mainly affected in the affected hemifield but not in the unaffected hemifield. More importantly, lower RPC density and thicker RNFL thickness were the features of BRVO affected eyes in the baseline, and RPC density can be a useful parameter to evaluate the perfusion status of ONH in BRVO.

## Figures and Tables

**Table 1:** Demographics and clinical characteristics of patients with unilateral BRVO.

Variables	Mean ± standard deviation
No. of patients	48
Age, y, mean ± SD	54.0 ± 9.50
Sex, male/female	22/26
Hypertension, *n* (%)	16(33.3%)
IOP of BRVO eyes, mmHg	15.6 ± 2.6
IOP of fellow eyes, mmHg	15.8 ± 2.5
BCVA of BRVO eyes	0.686 ± 0.424
BCVA of fellow eyes	0.131 ± 0.169
No. of superior/inferior	35/13

BRVO: branch retinal vein occlusion; IOP: intraocular pressure; BCVA: best-corrected visual acuity; superior/inferior: the eye with superior vein occlusion/the eye with inferior vein occlusion.

**Table 2:** The vessel density, RNFL thickness, and ONH analysis of unilateral BRVO.

OCTA parameters	BRVO eye	Fellow eye	*P* value
Section density (%)			
(i) WI capillary	49.18 ± 2.92	50.45 ± 2.52	0.006
(ii) WI all vessel	55.87 ± 2.83	56.83 ± 2.55	0.033
(iii) WI larger blood vessel	6.69 ± 0.69	6.38 ± 0.66	0.003
(iv) ID capillary	47.74 ± 5.2	46.79 ± 5.15	0.162
(v) ID all vessel	58.38 ± 4.47	57.48 ± 4.59	0.183
(vi) ID larger blood vessels	10.64 ± 1.90	10.69 ± 2.21	0.859
(vii) PP capillary	51.74 ± 3.45	54.1 ± 2.75	<0.001
(viii) PP all vessel	58.12 ± 3.36	60.14 ± 2.70	<0.001
(ix) PP larger blood vessels	6.37 ± 0.76	6.04 ± 0.69	0.019
RNFL thickness, um			
(i) PP	128.67 ± 23.72	118.13 ± 13.04	0.001
ONH analysis			
(i) Cup/disc area ratio	0.335 ± 0.164	0.336 ± 0.161	0.937
(ii) Cup/disc vertical ratio	0.526 ± 0.163	0.518 ± 0.180	0.590
(iii) Cup/disc horizontal ratio	0.625 ± 0.178	0.623 ± 0.200	0.915
(iv) Rim area (mm^2^)	1.492 ± 0.384	1.472 ± 0.354	0.52
(v) Cup volume (mm^3^)	0.169 ± 0.140	0.178 ± 0.169	0.517

RNFL: retinal nerve fiber layer thickness; ONH: optic nerve head; BRVO: branch retinal vein occlusion; OCTA: optical coherence tomography angiography; WI: whole image of 4.5 × 4.5 mm of Angio Disc scan; ID: inside disc; PP: peripapillary. All data are presented as mean ± SD, *P* < 0.05 was considered statistically significant.

**Table 3:** The vessel density of superior BRVO.

Section density (%)	BRVO eye	Fellow eye	*P* value
S-Hemi capillary	50.55 ± 4.34	54.27 ± 3.05	<0.001
S-Hemi all vessel	57.46 ± 3.98	60.58 ± 2.90	<0.001
S-Hemi larger blood vessel	6.91 ± 0.83	6.31 ± 0.87	0.003
I-Hemi capillary	53.84 ± 3.05	553.60 ± 3.09	0.629
I-Hemi all vessel	59.75 ± 2.86	59.28 ± 2.96	0.347
I-Hemi larger blood vessel	5.91 ± 0.74	5.69 ± 0.80	0.193
NS capillary	50.01 ± 5.26	50.77 ± 4.28	0.475
NI capillary	49.24 ± 4.78	48.46 ± 4.70	0.254
IN capillary	53.51 ± 4.02	54.5 ± 3.44	0.152
IT capillary	60.29 ± 4.69	59.71 ± 4.87	0.535
TI capillary	53.94 ± 4.97	53.58 ± 4.59	0.698
TS capillary	53.86 ± 4.55	56.94 ± 3.68	<0.001
ST capillary	49.24 ± 6.29	57.80 ± 4.13	<0.001
SN capillary	48.87 ± 5.91	53.45 ± 3.65	<0.001
S capillary	49.09 ± 5.42	55.56 ± 3.54	<0.001
N capillary	49.77 ± 4.00	49.79 ± 4.13	0.963
I capillary	56.56 ± 3.82	56.88 ± 3.72	0.641
T capillary	53.85 ± 4.16	55.49 ± 3.65	0.045
G11 all vessel	53.32 ± 5.30	54.79 ± 5.16	0.301
G12 all vessel	58.46 ± 4.31	61.00 ± 3.89	0.003
G13 all vessel	54.17 ± 4.76	56.59 ± 6.66	0.128
G21 all vessel	54.75 ± 4.41	54.87 ± 4.22	0.916
G22 all vessel	59.21 ± 4.05	58.07 ± 4.26	0.148
G23 all vessel	55.07 ± 3.41	54.89 ± 4.61	0.853
G31 all vessel	54.38 ± 6.97	53.38 ± 6.89	0.641
G32 all vessel	62.44 ± 3.92	61.60 ± 4.41	0.253
G33 all vessel	54.78 ± 5.87	55.09 ± 7.63	0.881

BRVO: branch retinal vein occlusion; S-Hemi: superior hemifield; I-Hemi: inferior hemifield; S: superior quadrant; T: temporal quadrant; I: inferior quadrant; N: nasal quadrant; NS: nasal superior sector; NI: nasal inferior sector; IN: inferior nasal sector; IT: inferior temporal sector; TI: temporal inferior sector; TS: temporal superior sector; ST: superior temporal sector; SN: superior nasal sector. G11-G33: the 4.5 × 4.5 mm area was divided into 9 equal parts, the three cells in the first row are named G11, G12, and G13; the three cells in the second row are named G21, G22, and G23; the three cells in the third row are named G31, G32, and G33. All data are presented as mean ± SD, *P* < 0.05 was considered statistically significant.

**Table 4:** The RNFL thickness of superior BRVO.

RNFL thickness, um	BRVO eye	Fellow eye	*P* value
S-Hemi	135.03 ± 35.79	119.65 ± 16.21	0.019
I-Hemi	126.88 ± 17.59	116.47 ± 13.22	<0.001
NS	123.29 ± 39.01	113.03 ± 24.78	0.074
NI	103.18 ± 27.68	89.88 ± 17.65	<0.001
IN	155.91 ± 24.52	146.85 ± 24.44	0.004
IT	159.82 ± 24.65	155.12 ± 26.00	0.271
TI	89.97 ± 31.35	75.97 ± 11.97	0.008
TS	114.24 ± 79.88	79.48 ± 10.72	0.018
ST	159.27 ± 58.16	142.06 ± 20.30	0.116
SN	153.88 ± 31.06	149.03 ± 24.97	0.368
S	154.56 ± 39.70	145.94 ± 19.12	0.260
N	112.68 ± 34.14	102.77 ± 20.70	0.034
I	154.79 ± 26.84	150.29 ± 21.9	0.189
T	103.00 ± 56.08	77.73 ± 10.40	0.013

BRVO: branch retinal vein occlusion; RNFL: retinal nerve fiber layer thickness; S-Hemi: superior hemifields; I-Hemi: inferior hemifields; S: superior quadrant; T: temporal quadrant; I: inferior quadrant; N: nasal quadrant; NS: nasal superior sector; NI: nasal inferior sector; IN: inferior nasal sector; IT: inferior temporal sector; TI: temporal inferior sector; TS: temporal superior sector; ST: superior temporal sector; SN: superior nasal sector. All data are presented as mean ± SD, *P* < 0.05 was considered statistically significant.

**Table 5:** The vessel density of inferior BRVO.

Section density (%)	BRVO eye	Fellow eye	*P* value
S-Hemi capillary	53.70 ± 3.57	54.77 ± 2.95	0.361
S-Hemi all vessel	59.62 ± 3.38	61.16 ± 2.92	0.194
S-Hemi larger blood vessel	5.92 ± 0.98	6.39 ± 0.90	0.114
I-Hemi capillary	46.99 ± 3.90	54.35 ± 2.26	<0.001
I-Hemi all vessel	53.68 ± 4.47	60.17 ± 2.01	0.001
I-Hemi larger blood vessel	6.69 ± 2.08	5.83 ± 0.83	0.225
NS capillary	49.78 ± 3.89	50.46 ± 4.89	0.673
NI capillary	49.88 ± 5.12	49.50 ± 4.36	0.766
IN capillary	45.98 ± 5.92	54.19 ± 4.09	0.001
IT capillary	44.77 ± 5.23	61.09 ± 2.55	<0.001
TI capillary	47.33 ± 6.01	54.40 ± 2.48	0.005
TS capillary	56.80 ± 4.95	59.49 ± 2.56	0.121
ST capillary	55.82 ± 5.15	58.26 ± 3.73	0.200
SN capillary	54.16 ± 3.30	52.35 ± 6.09	0.258
S capillary	54.64 ± 3.70	55.18 ± 4.31	0.720
N capillary	49.27 ± 4.36	50.09 ± 4.30	0.568
I capillary	45.27 ± 5.18	57.27 ± 3.32	<0.001
T capillary	52.36 ± 5.10	57.09 ± 2.39	0.018
G11 vessel	55.35 ± 5.15	54.25 ± 6.67	0.730
G12 vessel	60.59 ± 1.89	60.93 ± 4.47	0.782
G13 vessel	54.56 ± 4.69	57.06 ± 4.81	0.242
G21 vessel	53.74 ± 5.69	55.18 ± 3.28	0.522
G22 vessel	56.58 ± 4.98	58.06 ± 4.96	0.321
G23 vessel	55.48 ± 4.54	56.24 ± 5.10	0.724
G31 vessel	51.83 ± 4.48	54.26 ± 5.73	0.321
G32 vessel	53.65 ± 6.63	63.61 ± 3.48	<0.001
G33 vessel	52.23 ± 3.30	55.40 ± 5.59	0.105

BRVO: branch retinal vein occlusion; S-Hemi: superior hemifields; I-Hemi: inferior hemifields; S: superior quadrant; T: temporal quadrant; I: inferior quadrant; N: nasal quadrant; NS: nasal superior sector; NI: nasal inferior sector; IN: inferior nasal sector; IT: inferior temporal sector; TI: temporal inferior sector; TS: temporal superior sector; ST: superior temporal sector; SN: superior nasal sector. G11-G33: the 4.5 × 4.5 mm area was divided into 9 equal parts, the three cells in the first row are named G11, G12, and G13; the three cells in the second row are named G21, G22, and G23; the three cells in the third row are named G31, G32, and G33. All data are presented as mean ± SD, *P* < 0.05 was considered statistically significant.

**Table 6:** The RNFL thickness of inferior BRVO.

RNFL thickness, um	BRVO eye	Fellow eye	*P* value
S-Hemi	125.64 ± 20.11	118.36 ± 12.91	0.145
H-Hemi	116.36 ± 28.79	118.09 ± 14.29	0.777
NS	117.09 ± 19.22	112.09 ± 17.96	0.228
NI	99.36 ± 28.78	92.36 ± 18.20	0.315
IN	123.09 ± 38.91	152.45 ± 32.03	0.004
IT	141.18 ± 60.97	152.64 ± 18.87	0.550
TI	106.64 ± 40.52	77 ± 10.31	0.037
TS	107.00 ± 45.07	81.82 ± 5.88	0.087
ST	138.09 ± 27.84	131.64 ± 20.54	0.258
SN	146.55 ± 23.82	152.55 ± 26.98	0.416
S	142.55 ± 23.49	143.09 ± 19.30	0.911
N	109.36 ± 20.16	103.27 ± 17.44	0.136
I	131.09 ± 45.50	152.46 ± 21.07	0.059
T	106.82 ± 37.18	79.36 ± 6.50	0.032

BRVO: branch retinal vein occlusion: RNFL: retinal nerve fiber layer thickness; S-Hemi: superior hemifields; I-Hemi: inferior hemifields; S: superior quadrant; T: temporal quadrant; I: inferior quadrant; N: nasal quadrant; NS: nasal superior sector; NI: nasal inferior sector; IN: inferior nasal sector; IT: inferior temporal sector; TI: temporal inferior sector; TS: temporal superior sector; ST: superior temporal sector; SN: superior nasal sector. All data are presented as mean ± SD, *P* < 0.05 was considered statistically significant.

## Data Availability

Raw data were generated using OCTA (RTVue-XR Avanti, Optovue, Inc., Fremont, CA). Derived data supporting the findings of this study are available from the corresponding author Bojun Zhao on request.
